# Phenotypic and genotypic characterization of antibiotic resistance in the methicillin-resistant *Staphylococcus aureus* strains isolated from hospital cockroaches

**DOI:** 10.1186/s13756-019-0505-7

**Published:** 2019-03-13

**Authors:** Zohreh Abdolmaleki, Zohreh Mashak, Farhad Safarpoor Dehkordi

**Affiliations:** 1 Department of Pharmacology, Faculty of Veterinary Medicine, Karaj Branch, Islamic Azad University, Karaj, Iran; 2 Department of Food Hygiene, Faculty of Veterinary Medicine, Karaj Branch, Islamic Azad University, Karaj, Iran; 3 Halal Research Center of IRI, FDA, Tehran, Iran

**Keywords:** Methicillin-resistant *Staphylococcus aureus*, Antibiotic resistance, Antibiotic resistance genes, Hospital cockroaches

## Abstract

**Background:**

Cockroaches are one of the most important and frequent insects responsible for harboring, transmission and dissemination of human pathogens in the hospital environment. The present research was done to study the phenotypic and genotypic characterization of antibiotic resistance in the Methicillin-resistant *Staphylococcus aureus* strains isolated from hospital cockroaches.

**Methods:**

Five-hundred and thirty *Periplanets americana* and *Blattella germanica* cockroaches were collected and their gut content and external washing samples were subjected to bacterial isolation. MRSA strains were subjected to disk diffusion and PCR amplification of antibiotic resistance genes.

**Results:**

Prevalence of MRSA strains in *P. americana* and *B. germanica* cockroaches were 52.77 and 43.33%, respectively. External washing samples of *P. americana* cockroaches had the highest prevalence of MRSA strains (59.57%). MRSA isolates of external washing samples harbored the highest prevalence of resistance against penicillin (100%), ceftaroline (100%), tetracycline (100%), gentamicin (83.33%) and trimethoprim-sulfamethoxazole (80.55%). MRSA strains isolated from gut content samples harbored the highest prevalence of resistance against penicillin (100%), ceftaroline (100%), tetracycline (100%), trimethoprim-sulfamethoxazole (80%) and gentamicin (73.33%). *BlaZ*, *aacA-D*, *tetK*, *msrA*, *dfrA*, *ermA*, *gyrA*, *grlA* and *rpoB* were the most commonly detected antibiotic resistance genes amongst the MRSA strains.

**Conclusions:**

The present investigation is the first report of the phenotypic and genotypic evaluation of antibiotic resistance in the MRSA strains isolated from *P. americana* and *B. germanica* hospital cockroaches. Hospital cockroaches are considered as a potential mechanical vector for MRSA strains.

## Introduction

There are several ways to increase the survival of microorganisms in the environment and their transmission to human population. Cockroaches are among the most common insects found in industrial and residential environments such as hospitals. Indeed, the species has been highly successful in exploiting niches within human habitation. In addition to being a persistent pest and owing to their omnivorous habit of feeding and indiscriminate deposition of fecal materials, cockroaches are well-known agents for harboring, transmission and dissemination of human pathogens, thereby representing a public health risk [1, 2].

There are two cockroach species commonly found infesting in domestic, industrial and residential environments, these are the German cockroach (*Blattella germanica* (*B. germanica*)) and the American cockroach (*Periplanets americana* (*P. americana*)) (Dictyoptera; Blattidae). *P. americana* is the largest shiny reddish brown peridomestic cockroaches measuring on average 4–5 cm in length, while *B. germanica* is light yellowish brown with length ranging from 1.0–2.5 cm [1, 2]. *B. germanica* are the most abundantly distributed cockroach species. *B. germanica* are more prevalent inside the home, while the *P. americana* are common around the home and associated with water drainage systems and water pipes. Both of them can disseminate through the sewage system especially in public places such as hospitals [1, 2].

Over 100 species of virulent and resistant bacteria have been isolated from or passed through cockroaches. Microbiological and epidemiological investigations reported that *Staphylococcus aureus* (*S. aureus*) has commonly been isolated from *P. americana* and *B. germanica* found in the public places and especially hospital environment [3, 4]. *S. aureus* is commonly found in nose and respiratory system and on the skin [5]. It is responsible for the occurrence of nosocomial and community-acquired infections, food-borne diseases and food poisoning [5].

*S. aureus* strains are usually resist against several types of antibiotics [6–9]. Nowadays, methicillin-resistant *S. aureus* (MRSA) has become a serious problem in hospitals [6–9]. Documented data revealed that about 50–70% of the *S. aureus* strains isolated from the hospital environment were MRSA [6–9]. MRSA strains are responsible for about 100,000 cases of infections with around 20% mortality rate each year in the United States [10]. High pathogenicity of MRSA strains, its high resistance to several types of antibiotics and its nosocomial aspects have increased the importance of isolation of MRSA from the hospital cockroaches. MRSA strains are believed to serve as important reservoirs of antimicrobial resistance genes which can transfer and integrate into the MRSA genome leading to the emergence of new and potentially more resistant strains [6–9]. Documented data revealed that presence of certain antibiotic resistance genes is responsible for occurrence of severe antibiotic resistance [6–9]. Reports showed the high presence of *rpoB*, *blaZ*, *mecA*, *aacA-D*, *tetK* and *tetM*, *ermA* and *msrA*, *linA*, *vatA* and *vatB*, *dfrA*, *gyrA* and *grlA* and *cat1* antibiotic resistance genes in the *S. aureus* strains caused severe occurrence of resistance against ansamycins, penicillins, methicillin, aminoglycosides, tetracyclines, macrolides–lincosamide-streptogramin B, lincosamides, streptogramins, folate inhibitors, fluoroquinolones and phenicols groups of antibiotics, respectively [6–9]. Reports of methicillin-resistant strains are challenging due to the large proportion of methicillin-resistant strains and increasing numbers of isolates reinforcing the need to revise their importance to hospital [6–9]. Therefore, screening of these elements is important for public health and despite the importance of such a screen, limited data are available for MRSA at the species level among the hospital cockroach samples.

MRSA strains have been tested in hospital cockroach samples to assess microbiological safety, sanitation conditions and finally epidemiological roles of these insects in survival and transmission of bacteria. High pathogenicity of MRSA strains and general weakness of hospitalized patients make it necessary to assess the presence of MRSA strains in hospital cockroach samples. Thus, the current research was done to study the phenotypic and genotypic properties of antibiotic resistance in the MRSA strains isolated from *P. americana* and *B. germanica* cockroaches in Iranian hospitals.

## Materials and methods

### Samples

The present descriptive study was conducted during 2016 and 2017 at the tertiary hospitals of the Tehran province, Iran. Five-hundred and thirty cockroaches were collected using sticky traps, vacuum cleaners and hand catch methods from human dwellings. Traps were manufactured according to the design of Reierson and Rust (1997) [11]. The traps were placed on the floor under beds, cupboards, wooden racks, and/or benches, for two consecutive days. Each trapped cockroach was placed in a sterile test tube before sending to the laboratory. After immobilization by freezing at 0 °C for 5 min, the species of cockroaches were identified under a dissecting microscope according to Harwood and James (1979) [12]. *P. americana* and *B. germanica* cockroaches were subjected to further parts of the study.

### Sample preparation and isolation of *S. aureus*

Body surface of cockroaches were washed with physiological saline after vortexing for 2 min and taken as a homogenate sample. Before gastrointestinal tract (GIT) dissection, each cockroach was decontaminated with 95% ethanol, and the residue of ethanol was removed with saline solution. The gut was dissected aseptically using sterile needles and washed with 5-mL normal saline solution. Caution was taken to reduce the number of cut off or break in the gut. One milliliter of each homogenate was inoculated separately into 9-mL of buffered peptone water (Merck, Germany) for primary enrichment, and incubated at 37 °C for 18–24 h. Samples were then inoculated in screw cap test tube containing nutrient broth (NB, Merck, Germany) for the isolation of *S. aureus*, and enriched overnight at 37 °C. A loopful of inoculum from the enriched culture was streaked on to Blood Agar (BA, Merck, Germany) and Mannitol Salt Agar (MSA, Merck, Germany) incubated at 37 °C for 24 h for the observation of hemolysis and mannitol fermentation, respectively. Colonies with beta-hemolysis reaction and mannitol fermentation were further identified on the basis of Gram staining, catalase activity, coagulated test (rabbit plasma), oxidase test, glucose O/F test, resistance to bacitracin (0.04 U), urease activity, nitrate reduction, phosphatase, deoxyribonuclease (DNase, Merck, Germany) test, voges-proskaver (Merck, Germany) test and carbohydrate (xylose, sucrose, trehalose and maltose, fructose, lactose, mannose) fermentation tests [13].

### Identification of methicillin-resistant *Staphylococcus aureus* strains

Cefoxitin (30 μg) and oxacillin (1 μg) susceptibility tests were used to distinguish the MRSA strains from *S. aureus* isolates of hospital cockroaches. All tests were performed using the guidelines of the Clinical and Laboratory Standards Institute (CLSI) [14].

MRSA isolates were identified another time using the PCR-based amplification of *mecA* gene. MRSA strains were sub-cultured on TSB media (Merck, Germany) and further incubated for 48 h at 37 °C. Genomic DNA was extracted from bacterial colonies using the DNA extraction kit (Thermo Fisher Scientific, St. Leon-Rot, Germany) according to manufacturer’s instruction. Purity (A260/A280) and concentration of extracted DNA were then checked (NanoDrop, Thermo Scientific, Waltham, MA, USA). The truth of the DNA was assessed on a 2% agarose gel stained with ethidium bromide (0.5 μg/mL) (Thermo Fisher Scientific, St. Leon-Rot, Germany).

The PCR reactions were performed in a total volume of 25 μL, including 1.5 mM MgCl2, 50 mM KCl, 10 mM Tris-HCl (pH 9.0), 0.1% Triton X-100, 200 μM dNTPs each (Thermo Fisher Scientific, St. Leon-Rot, Germany), 2.5 μL PCR buffer (10X), 2.5 mM of each primer *mecA1* (5′-ACGAGTAGATGCTCAATATAA-3′) and *mecA2* (5′-CTTAGTTCTTTAGCGATTGC-3′) (Gen Bank Accession Number NC_003923M, 293 bp), 1.5 U of Taq DNA polymerase (Thermo Fisher Scientific, St. Leon-Rot, Germany) and 5 μL of the extracted DNA template of the MRSA isolates. The PCR cycling conditions were as follows: initial denaturation at 94 °C for 3 min, followed by 30 cycles of 94 °C for 30 s, 60 °C for 30 s, and 72 °C for 30 s, followed by an extra cycle of annealing at 60 °C for 30 s, and a final extension at 72 °C for 5 min.

### Phenotypic evaluation of antibiotic resistance

Patterns of antimicrobial resistance of the MRSA strains isolated from hospital cockroaches were studied using the simple disk diffusion according to the Kirby-Baur disc diffusion technique. The Mueller–Hinton agar (Merck, Germany) medium was used for this purpose. Susceptibility of MRSA isolates were tested against several types of antibiotic groups including Penicillins (penicillin (10 μg/disk)), Cephems (ceftaroline (30 μg/disk),), Aminoglycosides (gentamicin (10 μg/disk), amikacin (30 μg/disk), Macrolides (azithromycin (15 μg/disk) and erythromycin (15 μg/disk)), Tetracyclines (tetracycline (30 μg/disk), doxycycline (30 μg/disk)), Fluoroquinolones (ciprofloxacin (5 μg/disk) and levofloxacin (5 μg/disk)), Lincosamides (clindamycin (2 μg/disk)), Folate pathway inhibitors (trimethoprim-sulfamethoxazole (25 μg/disk)), Phenicols (chloramphenicol (30 μg/disk)) and Ansamycins (rifampin (5 μg/disk)) antibiotic agents (Oxoid, UK) using the instruction of Clinical and Laboratory Standards Institute [15]. The plates containing the discs were allowed to stand for at least 30 min before incubated at 37 °C for 24 h. The diameter of the zone of inhibition produced by each antibiotic disc was measured and interpreted using the CLSI zone diameter interpretative standards [15]. *Staphylococcus aureus* ATCC 25923 was used as quality control organism in antimicrobial susceptibility determination.

### Genotypic evaluation of antibiotic resistance

Table 1 represents the list of primers and PCR conditions used for amplification of antibiotic resistance genes in the MRSA strains isolated from hospital cockroaches [16–22]. A programmable DNA thermo-cycler (Eppendorf Mastercycler 5330, Eppendorf-Nethel-Hinz GmbH, Hamburg, Germany) was used in all PCR reactions. Amplified samples were analyzed by electrophoresis (120 V/208 mA) in 2.5% agarose gel. The gel was stained with 0.1% ethidium bromide (0.4 μg/ml). The UVI doc gel documentation systems (Grade GB004, Jencons PLC, London, UK) was applied for analysis of images.

**Table 1:** Target genes, oligonucleotide primers and PCR conditions used for detection of antibiotic resistance genes in the MRSA strains isolated from hospital cockroaches (16–22)

Target gene	Primer sequence (5′-3′)	PCR product (bp)	PCR programs	PCR volume (50 μL)
*AacA-D*	F: TAATCCAAGAGCAATAAGGGC R: GCCACACTATCATAACCACTA	227	1 cycle: 94 ^0C^ ------------ 5 min. 25 cycle: 94 ^0C^ ------------ 60 s 55 ^0C^ ------------ 70 s 72 ^0C^ ------------ 60 s 1 cycle: 72 ^0C^ ------------ 10 min	5 μL PCR buffer 10X 1.5 mM Mgcl_2_ 200 μM dNTP (Fermentas) 0.5 μM of each primers F & R 1.25 U Taq DNA polymerase (Fermentas) 2.5 μL DNA template
*ermA*	F: AAGCGGTAAACCCCTCTGA R: TTCGCAAATCCCTTCTCAAC	190		
*tetK*	F: GTAGCGACAATAGGTAATAGT R: GTAGTGACAATAAACCTCCTA	360		
*tetM*	F: AGTGGAGCGATTACAGAA R: CATATGTCCTGGCGTGTCTA	158	1 cycle: 94 ^0C^ ------------ 6 min. 34 cycle: 95 ^0C^ ------------ 50 s 55 ^0C^ ------------ 70 s 72 ^0C^ ------------ 60 s 1 cycle: 72 ^0C^ ------------ 8 min	5 μL PCR buffer 10X 2 mM Mgcl_2_ 200 μM dNTP (Fermentas) 0.5 μM of each primers F & R 1.5 U Taq DNA polymerase (Fermentas) 5 μL DNA template
*vatA*	F: TGGTCCCGGAACAACATTTAT R: TCCACCGACAATAGAATAGGG	268		
*msrA*	F: GGCACAATAAGAGTGTTTAAAGG R: AAGTTATATCATGAATAGATTGTCCTGTT	940	1 cycle: 94 ^0C^ ------------ 6 min. 34 cycle: 95 ^0C^ ------------ 60 s 50 ^0C^ ------------ 70 s 72 ^0C^ ------------ 70 s 1 cycle: 72 ^0C^ ------------ 8 min	5 μL PCR buffer 10X 2 mM Mgcl_2_ 150 μM dNTP (Fermentas) 0.75 μM of each primers F & R 1.5 U Taq DNA polymerase (Fermentas) 3 μL DNA template
*vatB*	F: GCTGCGAATTCAGTTGTTACA R: CTGACCAATCCCACCATTTTA	136	1 cycle: 94 ^0C^ ------------ 6 min. 35 cycle: 95 ^0C^ ------------ 50 s 55 ^0C^ ------------ 70 s 72 ^0C^ ------------ 80 s 1 cycle: 72 ^0C^ ------------ 10 min	5 μL PCR buffer 10X 2 mM Mgcl_2_ 150 μM dNTP (Fermentas) 0.75 μM of each primers F & R 1.5 U Taq DNA polymerase (Fermentas) 3 μL DNA template
*linA*	F: GGTGGCTGGGGGGTAGATGTATTAACTGG R: GCTTCTTTTGAAATACATGGTATTTTTCGA	323	1 cycle: 94 ^0C^ ------------ 6 min. 30 cycle: 95 ^0C^ ------------ 60 s 57 ^0C^ ------------ 60 s 72 ^0C^ ------------ 60 s 1 cycle: 72 ^0C^ ------------ 10 min	5 μL PCR buffer 10X 2 mM Mgcl_2_ 150 μM dNTP (Fermentas) 0.75 μM of each primers F & R 1.5 U Taq DNA polymerase (Fermentas) 3 μL DNA template
*blaZ*	F: ACTTCAACACCTGCTGCTTTC R: TGACCACTTTTATCA CAACC	490	1 cycle: 94 ^0C^ ------------ 5 min. 30 cycle: 94 ^0C^ ------------ 20 s 60 ^0C^ ------------ 30 s 72 ^0C^ ------------ 90 s 1 cycle: 72 ^0C^ ------------ 5 min	5 μL PCR buffer 10X 2 mM Mgcl_2_ 150 μM dNTP (Fermentas) 0.75 μM of each primers F & R 1.5 U Taq DNA polymerase (Fermentas) 3 μL DNA template
*cat1*	F: AGTTGCTCAATGTACCTATAACC R: TTGTAATTCATTAAGCATTCTGCC	547	1 cycle: 94 ^0C^ ------------ 8 min. 32 cycle: 95 ^0C^ ------------ 60 s 55 ^0C^ ------------ 70 s 72 ^0C^ ------------ 2 min 1 cycle: 72 ^0C^ ------------ 8 min	5 μL PCR buffer 10X 2 mM Mgcl_2_ 150 μM dNTP (Fermentas) 0.75 μM of each primers F & R 1.5 U Taq DNA polymerase (Fermentas) 3 μL DNA template
*gyrA*	F: AATGAACAAGGTATGACACC R: TACGCGCTTCAGTATAACGC	223	1 cycle: 94 ^0C^ ------------ 10 min. 25 cycle: 94 ^0C^ ------------ 20 s 52 ^0C^ ------------ 20 s 72 ^0C^ ------------ 50 s 1 cycle: 72 ^0C^ ------------ 5 min	5 μL PCR buffer 10X 2 mM Mgcl_2_ 150 μM dNTP (Fermentas) 0.75 μM of each primers F & R 1.5 U Taq DNA polymerase (Fermentas) 3 μL DNA template
*grlA*	F: ACTTGAAGATGTTTTAGGTGAT R: TTAGG AAATCTTGATGGCAA	459		
*dfrA*	*F: CTCACGATAAACAAAGAGTCA* *R: CAATCATTGCTTCGTATAACG*	201	1 cycle: 94 ^0C^ ------------ 2 min. 30 cycle: 94 ^0C^ ------------ 60 s 50 ^0C^ ------------ 60 s 72 ^0C^ ------------ 60 s 1 cycle: 72 ^0C^ ------------ 5 min	5 μL PCR buffer 10X 2 mM Mgcl_2_ 150 μM dNTP (Fermentas) 0.75 μM of each primers F & R 1.5 U Taq DNA polymerase (Fermentas) 3 μL DNA template
*rpoB*	F: ACCGTCGTTTACGTTCTGTA R: TCAGTGATAGCATGTGTATC	460	1 cycle: 94 ^0C^ ------------ 5 min. 32 cycle: 94 ^0C^ ------------ 60 s 56 ^0C^ ------------ 45 s 72 ^0C^ ------------ 60 s 1 cycle: 72 ^0C^ ------------ 10 min	5 μL PCR buffer 10X 2 mM Mgcl_2_ 150 μM dNTP (Fermentas) 0.75 μM of each primers F & R 1.5 U Taq DNA polymerase (Fermentas) 3 μL DNA template

### Statistical analysis

Statistical analysis was done using the SPSS 21.0 statistical software (SPSS Inc., Chicago, IL, USA). Chi-square test and Fisher’s exact two-tailed test were used to assess any significant relationship between prevalence of *S. aureus* and MRSA strains and their phenotypic and genotypic properties of antibiotic resistance. *P* value < 0.05 was considered as statistical significant level.

## Results

Table 2 represents the prevalence of *S. aureus* and MRSA strains in different types of hospital cockroaches. Sixty-five out of 530 (12.26%) external washing samples of hospital cockroaches and thirty-seven out of 530 (6.98%) gut content samples of hospital cockroaches were positive for *S. aureus* strains. Prevalence of *S. aureus* strains in *P. americana* and *B. germanica* hospital cockroaches were 11.61% (72/620) and 6.81% (30/440), respectively. External washing samples of *P. americana* hospital cockroaches had the highest prevalence of *S. aureus* strains (15.16%), while gut content samples of *P. americana* hospital cockroaches had the lowest (8.06%). Prevalence of *S. aureus* strains in external washing and gut content samples of *B. germanica* hospital cockroaches were 8.18 and 5.45%, respectively. Statistically significant differences were seen between types of hospital cockroaches and prevalence of *S. aureus* (*P* < 0.05).

**Table 2:** Prevalence of *S. aureus* and MRSA strains in different types of hospital cockroaches

Types of cockroaches		No samples collected	*N* (%) of *S. aureus* positive samples	N (%) of MRSA positive samples
*P. americana*	External washing	310	47 (15.16)	28 (59.57)
	Gut contents	310	25 (8.06)	10 (40)
*B. germanica*	External washing	220	18 (8.18)	8 (44.44)
	Gut contents	220	12 (5.45)	5 (41.66)
Total	External washing	530	65 (12.26)	36 (55.38)
	Gut contents	530	37 (6.98)	15 (40.54)

**Fig. 1:**
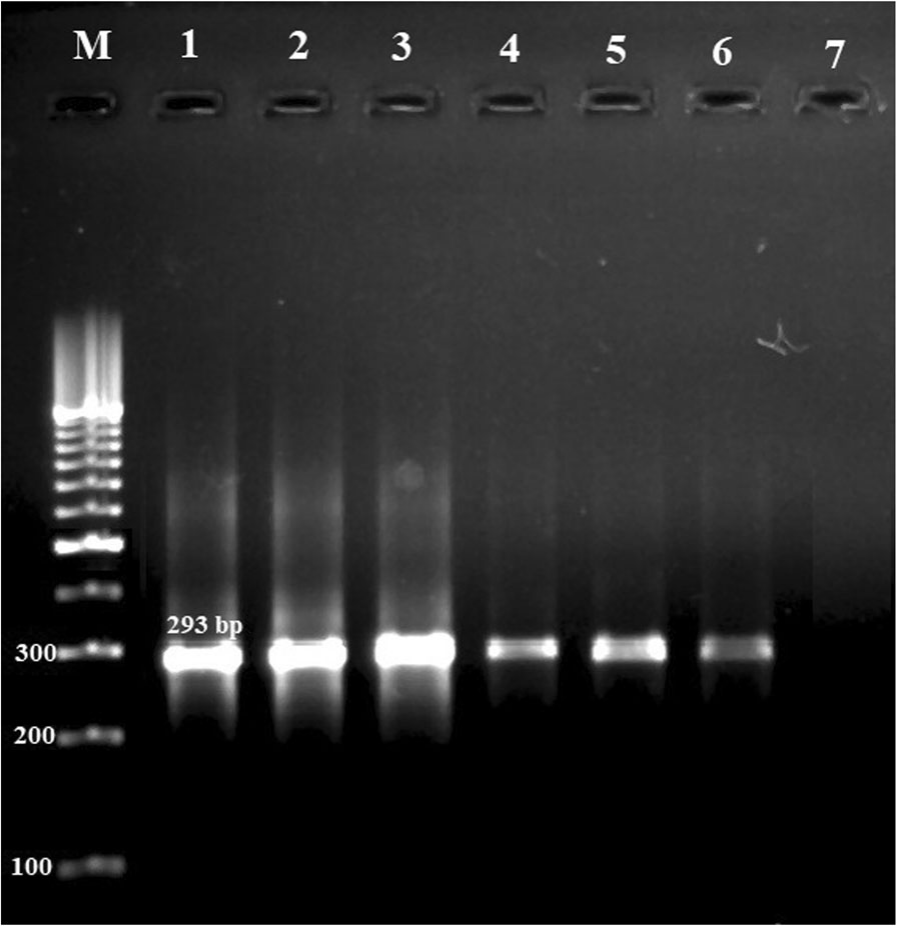
Gel electrophoresis of the *mecA* gene of the MRSA strains in PCR reaction. M: 100 bp ladder (Thermo Fisher Scientific, St. Leon-Rot, Germany), Lane 1: Positive control (MRSA ATCC 43300) Lanes 2–6: Positive samples for the *mecA* gene (293 bp) and Lane 7: Negative control (PCR grade water (Thermo Fisher Scientific, St. Leon-Rot, Germany))

MRSA strains were further identified using the *mecA* gene PCR amplification. Figure 1 shows the gel electrophoresis of the *mecA* gene of the MRSA strains in PCR reaction. Thirty-six out of 65 (55.38%) *S. aureus* strains isolated from external washing samples of hospital cockroaches and fifteen out of 37 (40.54%) *S. aureus* strains isolated from gut content samples of hospital cockroaches were confirmed to be MRSA strains. Prevalence of MRSA strains in *P. americana* and *B. germanica* hospital cockroaches were 52.77% (38/72) and 43.33% (13/30), respectively. External washing samples of *P. americana* hospital cockroaches had the highest prevalence of MRSA strains (59.57%), while gut content samples of *P. americana* hospital cockroaches had the lowest (40%). Prevalence of MRSA strains in external washing and gut content samples of *B. germanica* hospital cockroaches were 44.44 and 41.66%, respectively. Statistically significant differences were seen between types of hospital cockroaches and prevalence of MRSA (*P* < 0.05).

MRSA isolates were subjected to disk diffusion to assess the antibiotic resistance pattern. Table 3 represents the phenotypic pattern of antibiotic resistance of MRSA strains isolated from hospital cockroaches. MRSA strains isolated from external washing samples of hospital cockroaches harbored the highest prevalence of resistance against penicillin (100%), ceftaroline (100%), tetracycline (100%), gentamicin (83.33%) and trimethoprim-sulfamethoxazole (80.55%) antibiotic agents. MRSA strains isolated from gut content samples of hospital cockroaches harbored the highest prevalence of resistance against penicillin (100%), ceftaroline (100%), tetracycline (100%), trimethoprim-sulfamethoxazole (80%) and gentamicin (73.33%) antibiotic agents. MRSA strains isolated from external washing samples of hospital cockroaches harbored the lowest prevalence of resistance against chloramphenicol (25%), rifampin (33.33%), levofloxacin (38.88%) and ciprofloxacin (44.44%) antibiotic agents. MRSA strains isolated from gut content samples of hospital cockroaches harbored the lowest prevalence of resistance against chloramphenicol (20%), rifampin (33.33%), levofloxacin (40%), ciprofloxacin (46.66%) and clindamycin (46.66%) antibiotic agents. MRSA strains isolated from *P. americana* harbored the higher prevalence of resistance against almost all antibiotic agents (*P* < 0.05). Additionally, those of external washing samples had the higher prevalence of resistance against almost all tested antibiotic agents (*P* < 0.05). Statistically significant differences were seen between types of samples and prevalence of antibiotic resistance (*P* < 0.05).

**Table 3:** Phenotypic pattern of antibiotic resistance of MRSA strains isolated from different types of hospital cockroaches

Origins (*N* of MRSA strains)		*N* (%) isolates resistant to each antibiotic													
		Penicillins	Cephems	Aminoglycosides		Macrolides		Tetracyclines		Fluoroquinolones		Lincosamides	Folate inhibitors	Phenicols	Ansamycins
		P10^a^	Cft	Gen	Amk	Azi	Ert	Tet	Dox	Cip	Lev	Clin	Tr-Sul	C30	Rif
*P. americana*	External washing (28)	28 (100)	28 (100)	24 (85.71)	21 (75)	15 (53.57)	18 (64.28)	28 (100)	21 (75)	12 (42.85)	11 (39.28)	14 (50)	23 (82.14)	7 (25)	9 (36)
	Gut contents (10)	10 (100)	10 (100)	8 (80)	7 (70)	5 (50)	6 (60)	10 (100)	6 (60)	5 (50)	4 (40)	5 (50)	8 (80)	2 (20)	3 (30)
*B. germanica*	External washing (8)	8 (100)	8 (100)	6 (75)	5 (62.50)	4 (50)	5 (62.50)	8 (100)	5 (62.50)	4 (50)	3 (37.50)	4 (50)	6 (75)	2 (25)	3 (37.50)
	Gut contents (5)	5 (100)	5 (100)	3 (60)	2 (40)	3 (60)	3 (60)	5 (100)	2 (40)	2 (40)	2 (40)	2 (40)	4 (80)	1 (20)	2 (40)
Total	External washing (36)	36 (100)	36 (100)	30 (83.33)	26 (72.22)	19 (52.77)	23 (63.88)	36 (100)	26 (72.22)	16 (44.44)	14 (38.88)	18 (50)	29 (80.55)	9 (25)	12 (33.33)
	Gut contents (15)	15 (100)	15 (100)	11 (73.33)	9 (60)	8 (53.33)	9 (60)	15 (100)	8 (53.33)	7 (46.66)	6 (40)	7 (46.66)	12 (80)	3 (20)	5 (33.33)

^a^P10: penicillin (10 μg/disk), Cft: ceftaroline (30 μg/disk), Gen: gentamicin (10 μg/disk), Amk: amikacin (30 μg/disk), Azi: azithromycin (15 μg/disk), Ert: erythromycin (15 μg/disk), Tet: tetracycline (30 μg/disk), Do: doxycycline (30 μg/disk), Cip: ciprofloxacin (5 μg/disk), Lev: levofloxacin (5 μg/disk), Clin: clindamycin (2 μg/disk), Tr-Sul: trimethoprim-sulfamethoxazole (25 μg/disk), C30: chloramphenicol (30 μg/disk), Rif: rifampin (5 μg/disk)

**Fig. 2:**
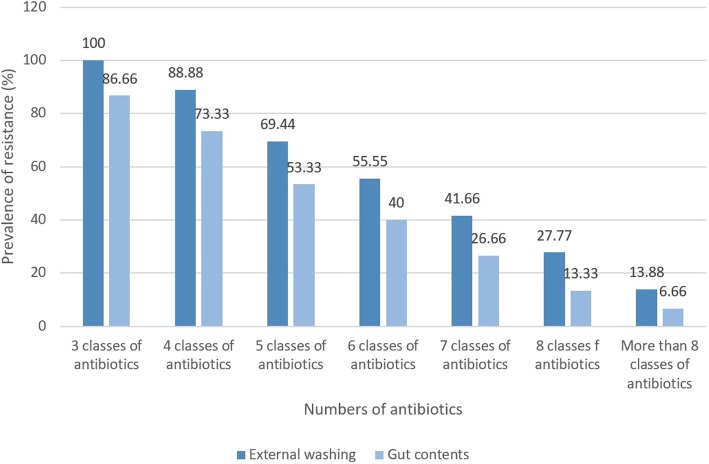
Prevalence of resistant MRSA strains in external washing and gut content samples of hospital cockroaches. Results were analyzed based on the total of 36 MRSA strains isolated from external washing and 15 MRSA strains isolated from gut content samples of hospital cockroaches

Figure 2 represents the prevalence of resistant MRSA strains in different types of hospital cockroaches. Multi-drug resistant strains were determined as those who had at least simultaneous resistance against 3 or more than 3 classes of antibiotics. All of the MRSA strains isolated from external washing samples of hospital cockroaches at least had resistance against 3 different classes of antibiotics, while prevalence of resistance against more than 8 classes of antibiotics was 13.88%. Additionally, 86.66% of the MRSA strains isolated from gut content samples of hospital cockroaches at least had resistance against 3 different classes of antibiotics, while prevalence of resistance against more than 8 classes of antibiotics was 6.66%.

**Fig. 3:**
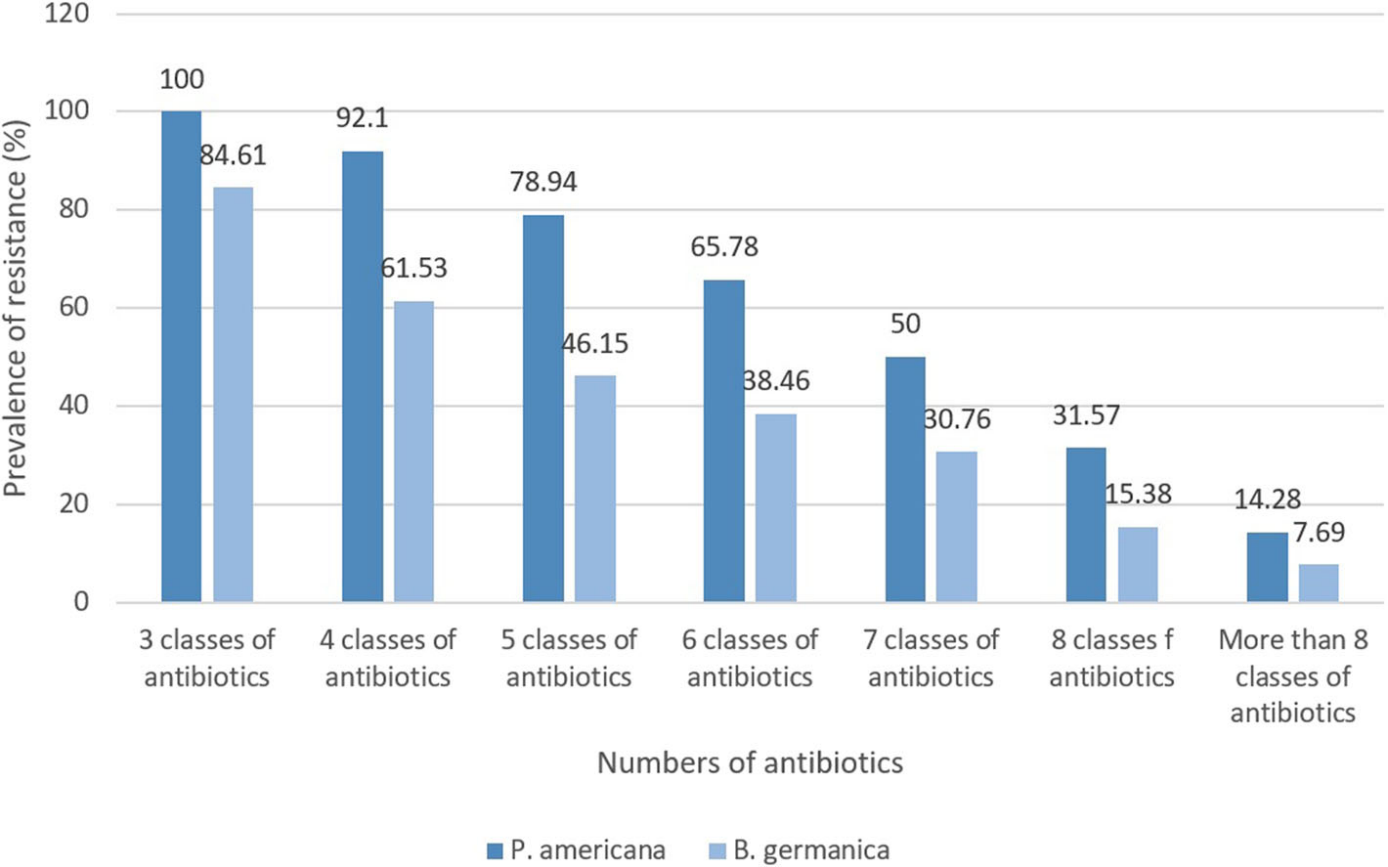
Prevalence of resistant MRSA strains in *P. americana* and *B. germanica* hospital cockroaches. Results were analyzed based on the total of 38 MRSA strains isolated from *P. americana* and 13 MRSA strains isolated from *B. germanica* hospital cockroaches

Figure 3 represents the prevalence of resistant MRSA strains in *P. americana* and *B. germanica* hospital cockroaches. All of the MRSA strains isolated from *P. americana* hospital cockroaches at least had resistance against 3 different classes of antibiotics, while prevalence of resistance against more than 8 classes of antibiotics was 14.28%. Additionally, 84.61% of the MRSA strains isolated from *B. germanica* hospital cockroaches at least had resistance against 3 different classes of antibiotics, while prevalence of resistance against more than 8 classes of antibiotics was 7.69%.

Table 4 represents the genotypic pattern of antibiotic resistance amongst the MRSA strain isolated from different types of hospital cockroaches. We found that *blaZ* (100%), *aacA-D* (88.88%), *tetK* (72.22%), *msrA* (63.88%), and *dfrA* (63.88%) were the most commonly detected antibiotic resistance genes amongst the MRSA strains isolated from external washing of hospital cockroaches. Additionally, *blaZ* (100%), *aacA-D* (73.33%), *msrA* (66.66%), *tetK* (60%) and *dfrA* (60%) were the most commonly detected antibiotic resistance genes amongst the MRSA strains isolated from gut contents of hospital cockroaches. We found that *vatB* (11.11%), *cat1* (19.44%), *tetM* (27.77%), *grlA* (27.77%), *rpoB* (27.77%) and *vatA* (30.55%) had the lowest distribution amongst the antibiotic resistance genes in MRSA strains isolated from external washing of hospital cockroaches. Additionally, *tetM* (20%), *linA* (20%), *ermA* (20%), *vatB* (20%), *grlA* (20%), *rpoB* (20%) and *cat1* (20%) had the lowest distribution amongst the antibiotic resistance genes in MRSA strains isolated from gut contents of hospital cockroaches. MRSA strains isolated from *P. americana* harbored the higher prevalence of antibiotic resistance genes (*P* < 0.05). Additionally, those of external washing had the higher prevalence of antibiotic resistance genes (*P* < 0.05). Statistically significant differences were seen between types of samples and prevalence of antibiotic resistance genes (*P* < 0.05).

**Table 4:** Genotypic pattern of antibiotic resistance amongst the MRSA strain isolated from different types of hospital cockroaches

Origins (N of MRSA strains)		N (%) isolates resistant to each antibiotic													
		Aminoglycosides	Tetracyclines		Macrolides		Lincosamides	Streptogramins		Penicillins	Folate inhibitors	Fluoroquinolones		Ansamycins	Phenicols
		*aacA-D*	*tetK*	*tetM*	*msrA*	*ermA*	*linA*	*vatA*	*vatB*	*blaZ*	*dfrA*	*gyrA*	*grlA*	*rpoB*	*cat1*
*P. americana*	External washing (28)	26 (92.85)	20 (71.42)	7 (25)	18 (64.28)	15 (53.57)	11 (39.28)	7 (25)	2 (7.14)	28 (100)	18 (64.28)	10 (35.71)	8 (28.57)	7 (25)	5 (17.85)
	Gut contents (10)	8 (80)	6 (60)	3 (30)	7 (70)	6 (60)	3 (30)	4 (40)	1 (10)	10 (100)	6 (60)	4 (40)	2 (20)	2 (20)	2 (20)
*B. germanica*	External washing (8)	6 (75)	6 (75)	3 (37.50)	5 (62.50)	3 (37.50)	2 (25)	4 (50)	2 (25)	8 (100)	5 (62.50)	4 (50)	2 (25)	3 (37.50)	2 (25)
	Gut contents (5)	3 (60)	3 (60)	1 (20)	3 (60)	1 (20)	1 (20)	3 (60)	1 (20)	5 (100)	3 (60)	3 (60)	1 (20)	1 (20)	1 (20)
Total	External washing (36)	32 (88.88)	26 (72.22)	10 (27.77)	23 (63.88)	18 (50)	13 (36.11)	11 (30.55)	4 (11.11)	36 (100)	23 (63.88)	14 (38.88)	10 (27.77)	10 (27.77)	7 (19.44)
	Gut contents (15)	11 (73.33)	9 (60)	4 (26.66)	10 (66.66)	7 (46.66)	4 (26.66)	7 (46.66)	2 (13.33)	15 (100)	9 (60)	7 (46.66)	3 (20)	3 (20)	3 (20)

## Discussion

Cockroaches are among the most notorious pests, having nocturnal and filthy habits, which do not only contaminate food by indiscriminate deposition of fecal materials and cause food poisoning but also transmit bacteria and other pathogenic microorganisms in infested areas. Cockroaches are common in many of human habitations, particularly in place where food is stored, processed, prepared, or served. Apart from that, they are also frequently detected in hospital environments, such as wards, operational rooms, area of intensive care units, and laboratory rooms. Indeed, cockroaches are found everywhere and possess nocturnal and omnivorous features; these characteristics make them the ideal carriers of pathogenic, virulent and resistant microorganisms and especially *S. aureus* [23].

The present investigation showed that 12.26% of external washing and 6.98% of gut content samples of hospital cockroaches were positive for *S. aureus* strains. Additionally, the prevalence of MRSA strains in external washing and gut content samples of hospital cockroaches were 6.79% (36/530) and 2.83% (15/530), respectively. Furthermore, *P. americana* cockroaches had the higher prevalence of *S. aureus* and also MRSA strains. Findings represents an important public health issue regarding the presence of *B. germanica* and *P. americana* cockroaches in hospital environment and also their high importance as a dangerous vector for survival and dissemination of *S. aureus* and also MRSA strains in the hospital environment. Findings also represent the higher prevalence of *S. aureus* and also MRSA strains in external washing samples of studied hospital cockroaches which may signify the higher role of external parts of the hospital cockroaches for dissemination of *S. aureus* and also MRSA strains. Moreover, *S. aureus* is not intestinal bacterium with high distribution in gut contents of the hospital cockroaches. However, presence of *S. aureus* in gut content samples of hospital cockroaches may be due to the permanent presence of *S. aureus* in hospital food samples [6, 7, 24] which may be consumed by cockroaches. Higher prevalence of *S. aureus* and MRSA in *P. americana* is may be due to their higher presence in hospital sewage than *B. germanica* cockroaches. Although *P. americana* is three to four folds larger than *B. germanica* in length, the capability of harboring microorganisms in cockroaches is not only related to their sizes but may also depend on the sanitation conditions of the environment.

*S. aureus* has been introduced as one of the most prevalent pathogenic bacteria isolated from the external washing and gut content samples of both *B. germanica* and *P. americana* cockroaches all-around the world [3, 4, 25–27]. Prevalence of *S. aureus* strains amongst the cockroach samples collected from Bangladesh [25], Iran [28], Nigeria [29], Ethiopia [1], Algeria [4], Brazil [30] and China [31] were 38, 62.86, 7.70, 69.20, 13.80, 49 and 5%, respectively.

Research on *S. aureus* carried by cockroaches in hospital environments is very important in relation to the control of nosocomial infections, which are becoming a major challenge. These infections cause high morbidity and mortality and increased hospitalization time having a consequent increase in costs. Cockroaches are able to explore various spaces in a hospital environment, making them a potential health risk, due to their ability to disperse pathogenic strains. Their growth is facilitated by fluids and food, as well as structural flaws in the hospital environment. Hospital infection is a major challenge for health professionals working in this area. The need to control and limit cockroaches has been stressed by most researchers.

MRSA strains isolated from hospital cockroaches harbored relatively high prevalence of resistance against tested antibiotic agents. MRSA strains harbored the highest prevalence of resistance against penicillin, ceftaroline, tetracycline, gentamicin, trimethoprim-sulfamethoxazole antibiotic agents. Additionally, prevalence of *blaZ*, *aacA-D*, *tetK*, *msrA* and *dfrA* was relatively higher than other tested antibiotic resistance genes. We found that phenotypic properties of antibiotic resistance were approved using the genotypic characterization of antibiotic resistance. MRSA strains isolated from *P. americana* cockroaches and also external washing samples had the higher prevalence of antibiotic resistance and antibiotic resistance genes. We also found the higher prevalence of resistance against human-based antibiotic agents in the MRSA strains isolated from hospital cockroaches which may indirectly show transmission of resistant MRSA strains from infected patients hospitalized in hospitals and health care units to resident cockroaches of hospitals. Antibiotics are not normally applied on cockroaches, but it is known that high resistance rates were reported among pathogens associated foods. In fact, a great association between cockroaches and foods could be the probable reason for isolation of resistant strains from cockroaches. Our findings revealed that more than 50% of *S. aureus* isolates were MRSA. High prevalence of antibiotic resistance reported in the present study is may be due to the unauthorized and indiscriminate prescription of antibiotic agents in Iranian hospitals and health care units. The circumstances in developing countries like Iran may be inflated by easy convenience of antimicrobials at a cheaper price and their extensive use in medicine.

According to the reports, burst of MRSA is increasing in Europe. In Austria, 21.60%; Belgium, 25.10%; Spain, 30.30%; and France, 33.60% of isolated *S. aureus* strains are methicillin resistant [32]. Higher pathogenicity and resistance of MRSA strains have also been reported in different types of human clinical infections [33, 34]. Similar antibiotic resistance patterns of the MRSA strains have also been reported against aminoglycosides [6–9, 35–38], cephems [6–9, 35–38], penicillins [6–9, 35–38], macrolides [35–38], tetracyclines [6–9, 36, 37], fluoroquinolones [6–9, 35–38], lincosamides [6–9, 35–38], folate inhibitors [6–9, 35–38], phenicols [6–9, 36, 37] and ansamycins [6–9, 36, 37] groups of antibiotics. Islam et al. (2016) [25] reported that *S. aureus* strains isolated from cockroach samples harbored the highest prevalence of resistance against erythromycin (58%), kanamycin (23%), penicillin (71%), oxacillin (45.50%), cephalothin (11%) and clindamycin (38%) antibiotic agents. Prado et al. (2006) [39] reported that *S. aureus* strains isolated from cockroach samples harbored the highest prevalence of resistance against ampicillin (30.80%), cephalexin (30.80%), cefepime (23%), and oxacillin (38.50%) antibiotic agents. Fowoyo and Ogunbanwo (2017) [40] reported that the *S. aureus* strains exhibited the high prevalence of resistance against ampicillin (86.70%), trimethoprim–sulfamethoxazole (74.90%), amoxicillin–clavulanic acid (52.50%), cefotaxime (3.50%), oxacillin (35.70%), ciprofloxacin (23.90%), erythromycin (15.70%), gentamicin (11.40%) and ofloxacin (7.10%). Rong et al. (2017) [41] reported that the prevalence of antibiotic resistance in the *S. aureus* strains against ampicillin, penicillin, amoxicillin–clavulanic acid, cefoxitin, ceftazidime, cefepime, kanamycin, streptomycin, amikacin, gentamicin, norfloxacin, ciprofloxacin, erythromycin, tetracycline, clindamycin, chloramphenicol, trimethoprim-sulfamethoxazole, vancomycin and rifampicin were 88.20, 88.20, 73.90, 8.40, 10.90, 8.40, 22.70, 14.30, 1.70, 4.20, 6.70, 5.00, 53.80, 26.90, 12.60, 7.50, 7.50, 0 and 2.50%, respectively.

We found that all of the MRSA strains were positive for the *mecA* gene. Most of the tetracycline-resistant MRSA isolates harbored *tetK* and *tetM* genes. Prevalence of *aacA-D* gene was high among gentamicin and amikacin-resistant MRSA strains. Prevalence of *msrA*, *ermA* and *linA* antibiotic resistance genes were also significant among the macrolide, erythromycin and clindamycin-resistant MRSA strains. Additionally, high prevalence of *dfrA*, *rpoB* and *cat1* antibiotic resistance genes were also found amongst the trimethoprim-sulfamethoxazole, rifampin and chloramphenicol-resistant MRSA strains. Finally, high distribution of *gyrA* and *grlA* antibiotic resistance genes were found amongst the ciprofloxacin and levofloxacin-resistant MRSA strains. Therefore, the pattern of the antibiotic resistance of the MRSA strains of hospital cockroach samples was confirmed by the PCR amplification of the specific antibiotic resistance genes. MRSA strains of our study had considerable prevalence of resistance against clindamycin (44.44 to 46.66%). The most imperative mechanism involving resistance against clindamycin is modulated by methylase enzyme which often encoded by *ermA* gene [42]. Prevalence of *ermA* antibiotic resistance genes among the MRSA strains of our research were 46.66 to 50%. Majority of our isolates carried two tetracyclines, two erythromycins, one macrolide and several streptogramin resistance determinants reveals a great diffusion of these types of resistance. *TetK*, *ermA*, *msrA* and *aacA-D* which encode resistance against tetracycline, erythromycin, macrolides and aminoglycosides were the most commonly detected antibiotic resistance genes in the MRSA strains isolated of hospital cockroach samples. Furthermore, prevalence of *blaZ*, *dfrA* and *gyrA* antibiotic resistance genes which encode resistance against penicillins, folate inhibitors and fluoroquinolones, respectively. The literature survey did not indicate any report on the prevalence of *blaZ*, *rpoB*, *gyrA*, *grlA*, *vatA*, *vatB*, *vatC*, *dfrA*, *cat1*, *msrA*, *ermA*, *linA*, *aacA-D*, *tetK* and *tetM* genes among the MRSA strains of hospital cockroach samples. Kumar et al. (2010) [43] reported that the most commonly identified antibiotic resistance genes among the *S. aureus* isolates were *linA* (51.60%), *msrB* (46.10%), *tetK* (34.40%), *tetM* (34.40%), *msrA* (26.60%) and *aacA-D* (26.60%). Karataş et al. (2017) [44] revealed the higher prevalence of *ermA* than *ermc* antibiotic resistance genes among the clindamycin, erythromycin and telithromycin-resistant and also higher prevalence of *tetM* than *tetK* antibiotic resistance genes among the tetracycline-resistant MRSA strains which were similar to our findings. Our results were also similar with those of the previous research which was conducted by Simeoni et al. (2008) [45]. They reported that the prevalence of *tetM*, *tetO*, *tetK*, *ermA*, *ermB*, *ermC*, *aac*, *blaZ* and *mecA* antibiotic resistance genes amongst the *S. aureus* strains isolated from meat samples were 100, 0, 91.66, 16.66, 33.33, 58.33, 0, 100 and 58.33%, respectively. High prevalence of *tetK* and *tetM* antibiotic resistance genes in the MRSA isolates can be clarified by their usual genetic locations. Presence of *tetK* gene on small multicopy plasmids and *tetM* on conjugative transposons contributes to the spread of these determinants [46]. Some of the MRSA strains harbored *ermA* gene. This gene is often located on small multicopy plasmids which are present in many different staphylococcal species [46]. The *ermA* gene is usually carried by transposons which could explain its high prevalence amongst the MRSA strains. Resistance to aminoglycosides (60 to 83.33%) which encode by the *aacA-D* gene (73.33 to 88.88%) is also prevalent. Johler et al. (2011) [46] reported that prevalence of *ermA*, *tetK* and *tetM* antibiotic resistance genes among the *S. aureus* strains isolated from cases of food poisoning, milk and pork were 25, 4.87 and 0%, 50, 0 and 12.82% and 0, 12.19, and 53.84%, respectively. Podkowik et al. (2012) [47] reported that the prevalence of tetracycline resistance genes (*tetO*, *tetK* and *tetM*) and erythromycin resistance methylase gene (*ermA*, *ermB* and *ermC*) among the *S. aureus* strains were 44 and 60%, respectively. Prevalence of *blaZ*, *rpoB*, *dfrA*, *gyrA*, *grlA* and *cat1* antibiotic resistance genes amongst the MRSA strains isolated from hospital cockroaches were 100%, 20–27.77%, 60–63.88%, 38.88–46.66%, 20–27.77% and 19.44–20%, respectively. High prevalence of *blaZ*, *rpoB*, *dfrA*, *gyrA*, *grlA* and *cat1* antibiotic resistance genes was also reported in the *S. aureus* strains isolated from human clinical infection samples [48–50]. Resistance to benzylpenicillin is mainly caused by the *blaZ* gene encoding production of beta-lactamases, which hydrolytically destroy beta-lactams. Our results suggest that *bla*Z may play a major role in occurrence of resistance against penicillins but cannot be used alone as an indicator for penicillin resistance. Rifampin acts by interacting specifically with the β subunit of the bacterial RNA polymerase encoded by the *rpoB* gene. *RpoB* expression will increase the prevalence of resistance against rifampin in the *S. aureus* strains. Low prevalence of cat1 antibiotic resistance gene is may be due to the low levels of prescription of chloramphenicol in veterinary and medical sciences. Additionally, chloramphenicol is forbidden antibiotic agent in Iran. We also found the high prevalence of MRSA strains in the hospital cockroach samples. The multi-drug resistant *S. aureus* in cockroach was reported earlier from Nigeria, Ethiopia, Iran, and Brazil [3, 4, 51–53]. Though this study tested multi-drug resistant against single bacterium, in the absence of prior work, the findings of this study would signify the emergence of MRSA in the environment and the prospective likelihood of dissemination of such strains through mechanical vector *B. germanica* and *P. americana* cockroaches in Iran.

MRSA strains are considered as important foodborne pathogens all-around the world. Thus, their high prevalence in hospital cockroaches may influence on their presence in food samples of hospitals. It is because of the fact that cockroaches can easily penetrate into the hospital food store units. High prevalence of *S. aureus* and other foodborne pathogens have also been reported previously [54-64].

## Conclusions

The present investigation is the first report of the phenotypic and genotypic evaluation of antibiotic resistance in the MRSA strains isolated from external washing and gut content samples of *B. germanica* and *P. americana* hospital cockroaches. High prevalence of *S. aureus* and MRSA strains, higher prevalence of bacteria in *P. americana* cockroaches, higher prevalence of bacteria in external washing samples of cockroaches, high prevalence of resistance against penicillin, ceftaroline tetracycline, gentamicin and trimethoprim-sulfamethoxazole, high distribution of *blaZ*, *aacA-D*, *tetK*, *msrA*, *dfrA*, *ermA*, *gyrA* and *grlA* antibiotic resistance genes and higher prevalence of MRSA strains in external washing samples of *P. americana* cockroaches were the most important findings of the present study. The present study shows the high importance of hospital cockroaches as dangerous reservoirs for harboring of virulent and resistant MRSA strains in the hospital environment and their transmission to human population. Otherwise, external surface of the *P. americana* cockroaches can act as a mechanical vector for transmission of MRSA strains in the hospital environment in Iran. However, further studies are required to find additional knowledge about the microbiological and epidemiological roles of the hospital cockroaches in survival and transmission of antibiotic-resistant bacteria.

## Abbreviations

*Blattella germanica*: *B. germanica*
MRSA: Methicillin-resistant *Staphylococcus aureus*
PCR: Polymerase Chain Reaction
*Periplanets americana*: *P. americana*
*S. aureus*: *Staphylococcus aureus*
SPSS: Statistical Package for the Social Sciences
